# Low-Order Non-Spatial Effects Dominate Second-Order Spatial Effects in the Texture Quantifier Analysis of 18F-FDG-PET Images

**DOI:** 10.1371/journal.pone.0116574

**Published:** 2015-02-25

**Authors:** Frank J. Brooks, Perry W. Grigsby

**Affiliations:** 1 Department of Radiation Oncology, Washington University School of Medicine, Saint Louis, Missouri, United States of America; 2 Division of Nuclear Medicine, Mallinckrodt Institute of Radiology, Saint Louis, Missouri, United States of America; 3 Department of Obstetrics and Gynecology, Washington University Medical Center, Saint Louis, Missouri, United States of America; 4 Alvin J. Siteman Cancer Center, Washington University Medical Center, Saint Louis, Missouri, United States of America

## Abstract

**Background:**

There is increasing interest in applying image texture quantifiers to assess the intra-tumor heterogeneity observed in FDG-PET images of various cancers. Use of these quantifiers as prognostic indicators of disease outcome and/or treatment response has yielded inconsistent results. We study the general applicability of some well-established texture quantifiers to the image data unique to FDG-PET.

**Methods:**

We first created computer-simulated test images with statistical properties consistent with clinical image data for cancers of the uterine cervix. We specifically isolated second-order statistical effects from low-order effects and analyzed the resulting variation in common texture quantifiers in response to contrived image variations. We then analyzed the quantifiers computed for FIGOIIb cervical cancers via receiver operating characteristic (ROC) curves and via contingency table analysis of detrended quantifier values.

**Results:**

We found that image texture quantifiers depend strongly on low-effects such as tumor volume and SUV distribution. When low-order effects are controlled, the image texture quantifiers tested were not able to discern only the second-order effects. Furthermore, the results of clinical tumor heterogeneity studies might be tunable via choice of patient population analyzed.

**Conclusion:**

Some image texture quantifiers are strongly affected by factors distinct from the second-order effects researchers ostensibly seek to assess via those quantifiers.

## Introduction

Position emission tomography (PET) using the 18F-fluorodeoxyglucose (FDG) radiotracer is an imaging modality well-established for the location and sizing of many tumor types. During the image reconstruction process, measured positron intensity typically is normalized such that, to within acceptable noise, zero image intensity (black) corresponds to zero tracer uptake and maximum image intensity (white) corresponds to maximal uptake. While false-color schemes are sometimes used to visualize FDG-PET images against those obtained via other modalities, the predominant scheme for mathematical analysis is linear, gray shading between the intensity extrema. The resulting gray level images of tumors often exhibit obvious heterogeneity, where some regions within the tumor appear much brighter than other intra-tumor regions. Because uptake of the glucose analog FDG correlates with metabolic activity, the motivation behind quantifying the observed image heterogeneity is that perhaps insight will be gained into the stark biological heterogeneity known to exist within tumors [1–3]. Here, biological heterogeneity commonly refers to a mixed phenotypic population of cells within the tumor. This diversification results from both genetic—e.g., numerous proliferation cycles with a relatively high per-cycle mutation rate—and non-genetic sources such as interaction with the local microenvironment [3]. The result of these integrated effects is a tumor with spatially varying cellular population, density and vascularization. These variations offer one feasible explanation as to why otherwise similar tumor types exhibit various degrees of invasiveness and treatment response. In short, it seems that increased heterogeneity confounds tailoring therapy to a specific tumor and thus is linked with poor prognosis [3]. For these reasons, development of a robust technique for quantifying observed heterogeneity in FDG-PET images might be an important opportunity for developing indicators of disease outcome.

A search of PubMed reveals a considerable and rapidly increasing interest in the implications of intra-tumor heterogeneity and how that heterogeneity might be measured via current imaging technology. Toward this end, there have been many recent proposals to use “texture quantifiers” applied to FDG-PET images as prognostic heterogeneity quantifiers [4–15]. Image texture refers to the perceptible pattern or grain of an image [16]; texture is the combined variation of a gray level distribution and the spatial arrangement of those levels. Any statistical descriptor of the gray level distribution itself—such as the variance, skewness or area under the cumulative histogram—is a “first-order” measure; it describes only the gray levels available but not at all where in the image those levels occur. A second-order measure contains information about the spatial arrangement of the available levels. A texture quantifier is thus a second-order statistical description of the probability that a given gray level occurs next to another. The occurrences of gray levels or groups of gray levels are tallied conveniently in matrices. Quantifiers based upon such gray level co-occurrence matrices (GLCMs) proposed by Haralick et al. [17] and related matrices [18–21] seem to be those most commonly employed in clinical studies of image heterogeneity. However, this application of image texture quantifiers specifically to FDG-PET images of typical tumors is fraught with difficulty.

Foremost, there is no consistent, unique mapping between a given visual feature and a given quantifier value [17]. In other words, there is no reliable correspondence between any single quantifier and exactly one, well-defined image feature. Thus, texture quantifiers may not be presumed to provide the categorization or objectivity sought when comparing tumors that are already difficult to describe verbally. Furthermore, there is no established scale for any texture quantifier applied to FDG-PET images. That is, there can be no presumption whatsoever that twice a quantifier value implies twice the heterogeneity. This immediately calls into question the increasingly common practice of comparing quantifier magnitudes and performing what amount to statistical location tests on pooled quantifier data. This practice is further complicated by the sheer number of quantifiers contrived and their latent inter-correlations which together yield far more degrees of freedom than the typical number of clinical dichotomies analyzed can support. In other words, having a computer algorithmically compute the gamut of GLCM-based texture quantifiers yields numerous predictors that are related in ways that might not be adequately testable via the relatively few number of clinical events observed in typical studies.

Equally as important, the minimum image sampling required for statistically robust analysis via texture quantifiers has not been established [22]. Consider that typical images for which image texture metics have proven to be useful indicators of visually perceptible features [17, 23–27] tend to be much more pixel-dense than those clinicians seek to analyze. To better appreciate this, note that a typical light microscopy image of a histology slide might have a pixel size on the order of 1 μm while a typical PET scanner yields pixels on the order of 1 mm. In practice, even very large tumors (say, 200 cm^3^) comprise only a few thousand FDG-PET pixels. This permits the logical possibility that small and large tumors are biologically identical but will measure to different heterogeneities solely because of their difference in size. It is also possible that any actual biological heterogeneities yield measurable image effects smaller than those yielded by differing size alone. In fact, it has been argued [22, 28]—and subsequently verified for various clinical data [15, 29]—that some texture quantifiers depend strongly upon tumor volume.

It should also be appreciated that the analysis technique described by Haralick et al. in 1973 and established in numerous subsequent image science publications is *not* generally implemented in current clinical research. Haralick et al. proposed that their quantifiers be applied to images that first have undergone “equal-probability quantizing” [17]. In short, this (now) well-known image processing technique stretches the gray level histogram to the entire available bit depth; the gray values are mapped based upon rank and number rather than magnitude. This histogram equalization has the effect of enhancing contrast near histogram maxima while reducing contrast near histogram minima [16]. Thus, histogram equalization can have the desirable effects of increasing the visual contrast within one tumor while reducing the variation between distinct tumors. Despite this, most published clinical texture analyses we found have little or no mention of gray level quantization [10, 12, 13]. Of those that do, what often is actually described seems to be only a rescaling of gray levels, not equalization of those levels [5, 6, 8, 9, 14, 15]. Furthermore, the rescaling thus employed has been arbitrary and widely varied. It also has been argued that choices made in resampling technique actually create spurious correlations between the maximum standard uptake value (SUV_max_) and some texture quantifiers [15].

Given the concerns described above, the suggestion [29] that heterogeneity of patient population contributes largely to the heterogeneity measured via texture quantifiers seems a plausible explanation of the inconsistent results of clinical image heterogeneity studies summarized excellently by Orlhac et al. [15]. Here, a heterogeneous population implies the more traditional, statisticians’ definition of a population comprising disparate sub-populations. We hypothesize that although image texture quantifiers are capable of measuring second-order variation in images, that variation is overwhelmed by low-order effects for the specific case of FDG-PET images of tumors. It is the purpose of the present study to: measure and compare low- and second-order effects of pertinent image variation, establish their impact upon clinical studies and describe a method for the robust application of image texture quantifiers to clinical FDG-PET data.

## Methods

### Clinical Image Data

Fully anonymized FDG-PET image data from a previous study [29] was used in the present work with waiver of informed consent as approved by the Washington University Human Research Protection Office. In brief, patients with newly diagnosed cervical cancer who underwent FDG-PET or FDG-PET/CT were staged clinically according to FIGO staging (AJCC 2002, 6th edition). The selection criteria for inclusion into this study were FIGO clinical stage IIb tumors and squamous cell histology. The raw FDG-PET data were scatter- and attenuation-corrected via software native to the scanner (Biograph 40 True Point Tomograph Scanner; Siemens). Images were reconstructed using ordered-subset expectation maximization (8 subsets; 4 iterations) and a gaussian smoothing filter of 4 mm in full width at half maximum was applied after reconstruction. The primary tumor evident in each FDG-PET image set was visually identified and then segmented using the rule that any image voxel with standard uptake value (SUV) greater than 40% of the maximum SUV is part of the tumor [30]. A veteran oncologist then made slight manual adjustments to the region to remove any obvious non-tumor voxels (e.g., bladder). These data were exported as a set of (*x*, *y*, *z*) coordinates, each with a single 15-bit grayscale image intensity corresponding to radioactivity in Bq/mL. For the one specific task of comparing the ensemble gray level distribution to that of the simulated images described below, we thresholded the clinical data at 50% of the maximum observed SUV. Patients were re-imaged approximately three months after completing curative-intent chemoradiotherapy. On those follow-up scans, 66 patients were found to have no detectable tumor while 19 had residual and/or new tumor.

### Simulated Image Data

We stress that we in no way attempted to simulate the PET process or model tumor growth. Instead, we sought only to render images consistent with clinical observation but with some purposefully controlled statistical properties. In order to isolate and study low- and second-order effects upon texture quantifiers, we created virtual tumor objects of drastically varying heterogeneity using the lumpy object model [31] as implemented in Python 2.7 computer code. In brief, a given number of three-dimensional, radially symmetric Gaussian functions with tunable radii and heights were first randomly centered within some field of view (FOV) and then superposed. We began with a 32 x 32 x 32 voxel blank background (i.e., gray level zero). We centered a spherical FOV with radius (F) chosen at random from the range [4,8] voxels. Looking forward to an upcoming thresholding step, this is FOV constraint was necessary to create a small, closed object near the center of the image. In order to first create an homogeneous object, we set the number of Gaussian lumps to 1 plus a variate from a Poisson distribution with mean 2. The height of each lump was set to 1.0 and the radius was chosen at random from the range [F/2, F] voxels. The lump functions were superposed at each image voxel. White noise of 2.5% of the voxel value was then added to each voxel. The entire virtual object was normalized and rescaled to the 8-bit gray scale. Any voxel below a threshold of half the maximum (255) was set to zero. Assuming a PET scanner voxel size of 64 mm^3^, the object was retained only if the volume (V) was within the observed range of typical (for cervical cancer) clinical data, [5, 205] cm^3^. We henceforth refer to this object as the “homogeneous object.” The homogeneous object is then stratified along the *z*-axis into sequential two-dimensional slices and output as 8-bit PNG images.

**Fig 1:**
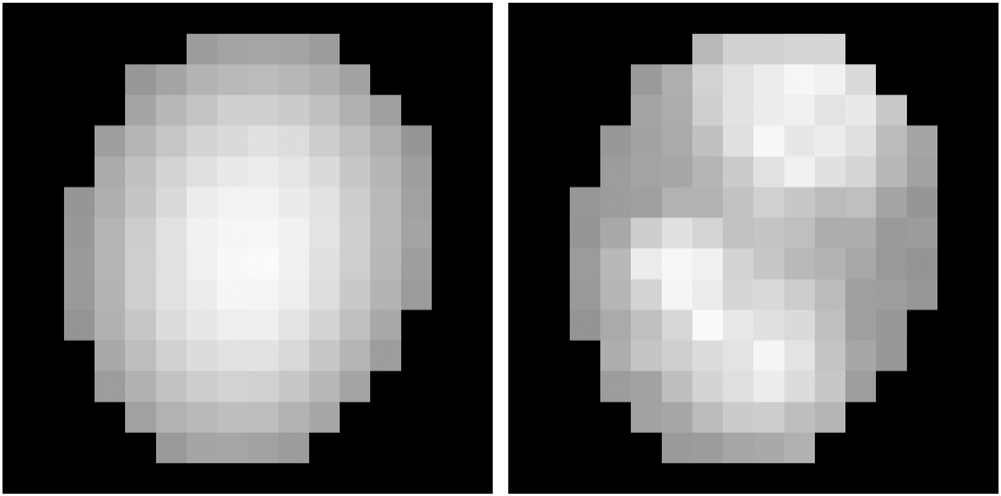
Cross-sections of simulated objects. Both three-dimensional objects have identical gray level distributions yet stark differences in spatial arrangement are clear. The vertical edge of the images corresponds to a length of 64 mm.

From the coordinates of the homogeneous object, a distinctly more heterogeneous object was created by adding more Gaussian lumps as follows. The number of lumps was set to V mod 5 cc. This yields a constant lump density across objects of widely varying size. Unique lump centers were chosen from the homogenous object. The lump heights were again set to 1.0 but the radii were chosen at random from the range [1, F/4] voxels. These smaller lumps were superposed for every homogeneous object voxel. To each voxel value, white noise of 5% of the voxel value and systematic white noise of 2% of the entire bit range were added. The resulting voxel values were rescaled to the same range as that of the homogeneous object, [127, 255]. In an effort to isolate second-order effects (arrangement) from first-order effects (distribution), we first performed histogram matching [32] of the heterogeneous object to that of the homogeneous object. Next, we used the histogram-matched heterogeneous object as a template for how the homogeneous gray levels should be arranged. This is done as follows. We first chose two homogeneous object coordinates at random. The total absolute difference in gray levels between the homogeneous object and the heterogeneous template object are computed using both coordinates. The gray levels at the two homogeneous coordinates are then swapped and the difference from the template object is recomputed. If the post-swap, inter-object difference is less than the pre-swap, inter-object difference, the swap is kept. This swapping process was repeated numerous times. Every 1000 swaps, the cross-correlation between the template and the swapped object was computed. When that cross-correlation became greater than 99%, swapping was halted. Because our image generation scheme is a stochastic process, it is possible, especially at very small volumes, that the heterogenous (swapped) object exhibits a very similar spatial arrangement to the homogeneous (unswapped) one. We therefore compute the cross-correlation between the homogeneous and heterogeneous objects. The pair is kept only if that correlation is less than 0.75. The heterogenous object is then stratified and saved. The result is a doublet of cross-sectional image sets of objects with distinctly different spatial arrangements but having *identical*: size, shape and gray level distributions. A typical example is given in Fig. 1 where the increased heterogeneity is obvious. We ran this image set generation process until we had 400 conjugate pairs. For the purposes of testing the sensitivity of texture quantifiers to spatial arrangement, it is also important to note that all of our generated objects are closed in the sense that they are smooth at the interface with the background and there are no holes within the object bulk. The ensemble distribution of object volume approximately follows a Gamma distribution with shape parameter 2.4 and scale parameter 29.3; the ensemble gray level distribution approximated that of our clinical data to within about 8% fractional error.

### Computation of Texture Quantifiers

For each image set (both clinical and simulated), the GLCM was computed as follows. An image set is read into memory as a set of (*x*, *y*, *z*, *g*) coordinates where (*x*, *y*, *z*) is the Cartesian coordinate of an object voxel and *g* > 0 is the gray level at that voxel. All object coordinates analyzed have been made available as anonymized supplementary data to this article. The gray levels are then non-linearly rescaled such that the gray level probability distribution is equalized to use the entire available bit depth [16, 17]. The gray level at a given coordinate and that at the (*x*_0_ + *i*, *y*_0_ + j, *z*_0_ + *k*) nearest neighbor of that coordinate form a gray level co-occurrence pair. The co-occurrences of all neighboring pairs are recorded in a distinct GLCM for each vector direction <i, *j*, *k*> [17]. Because we consider only object-to-object pairs where both members are nonzero, many of the possible directions yield redundant matrices. We therefore compute the GLCM for only <i, *j*, *k*> at the corners of the size one voxel first octant. The distinct GLCMs were individually normalized to create a set of symmetric matrices containing the probabilities that any one of the possible gray levels occurs at vector <i, *j*, *k*> from any other. It should be understood that many authors have renamed and redefined the quantifiers originally defined by Haralick et al. We follow the nomenclature found in the current medical literature and the definitions given in Ref. [16] as summarized in Table 1. It should be clear from these definitions that the homogeneity is inversely correlated with the dissimilarity. What may not be immediately clear to some, however, is that the energy and entropy are inverse correlates as well [16]. We chose these four quantifiers because they are widely implemented as FDG-PET image heterogeneity quantifiers yet have yielded inconsistent results. In our summations, we do not exhaustively tally every possible co-occurrence permitted by the given bit depth but instead count only co-occurrences between distinct gray levels actually present within the image. We computed a given texture quantifier on all eight directional GLCMs then averaged those values to obtain a single quantifier value for that set. This was repeated for each quantifier and then for each image set.

**Table 1:** Texture Quantifier Definitions.

Name	Alias	Formula
Angular Second Moment	Energy	$\sum_{m,n}p^{2}(m,n)$
Entropy		$-\sum_{m,n}p(m,n)ln\;p(m,n)$
Inverse Difference Moment	Homogeneity	$\sum_{m,n;m\neq n}\frac{p(m,n)}{\left|m-n\right|}$
Contrast	Dissimilarity	$\sum_{m,n}\left|m-n|\;p(m,n\right)$

Here, *p(m, n)* is the probability that distinct gray levels *m* and *n* appear in adjacency within the three-dimensional object analyzed.

### Statistical Analysis

For comparing gray-level histograms of entire image sets, we employed the two-sample Kolmogorov-Smirov test as computed in NumPy v1.8 (http://www.numpy.org). All other statistical analyses were conducted in R 3.1.0 (R Foundation for Statistical Computing, Vienna, Austria). Rank correlation between quantifier sets was assessed via Kendall’s τ. Correlation between dichotomized quantifier differences and binary treatment outcomes was assessed via Cramer’s *ϕ*. The prognostic potential of various texture quantifiers was assessed via receiver-operating curve (ROC) analysis as conducted via the pROC package [33].

## Results

### Simulated Image Data

**Fig 2:**
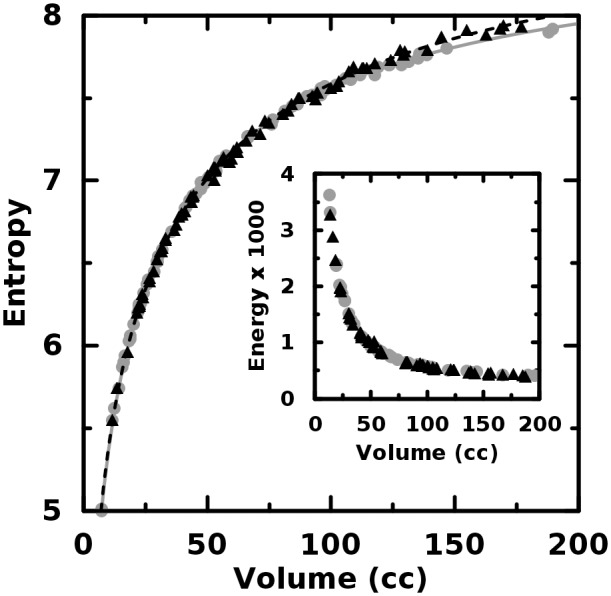
Entropy and energy plotted versus object volume. There is effectively no difference between these quantifiers for the homogeneous (gray, circles) and heterogeneous (black, triangles) objects.

We analyzed the simulated tumor images via the texture quantifiers given in Table 1. Fig. 2 shows the strong correlation between energy and entropy and the predicted strong dependence upon tumor volume. What is perhaps more important, however, is the lack of difference in quantifier scaling with volume between the homogenous objects (gray dots) and the heterogeneous objects (black triangles). For each GLCM-based quantifier analyzed, we computed the absolute percent difference between each conjugate pair using the homogeneous value as the normalizing value. The interquartile range (IQR) of the differences for the texture quantifiers given in Table 1 were: 2.3%, 0.26%, 8.0% and 8.2% respectively. Thus, in the case of the energy and the entropy, there is effectively no difference in value between the object pairs. Because each object pair differs only in spatial arrangement, the conclusion is that these two quantifiers are unable to distinguish purely second-order effects in image data consistent with that of clinical FDG-PET data. Additionally, those quantifiers are seen to be nearly perfect surrogates for tumor volume as Kendall’s *τ* = -0.97 and 0.98 for the energy and entropy, respectively.

**Fig 3:**
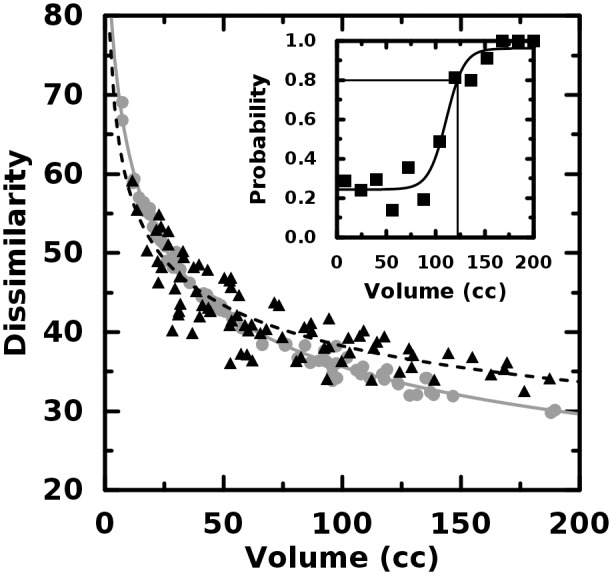
Dissimilarity plotted versus object volume. The dissimilarity scales *non-linearly* with volume. The trend curve for the homogeneous objects (gray, circles) crosses that of the heterogeneous objects (black, triangles).

**Fig 4:**
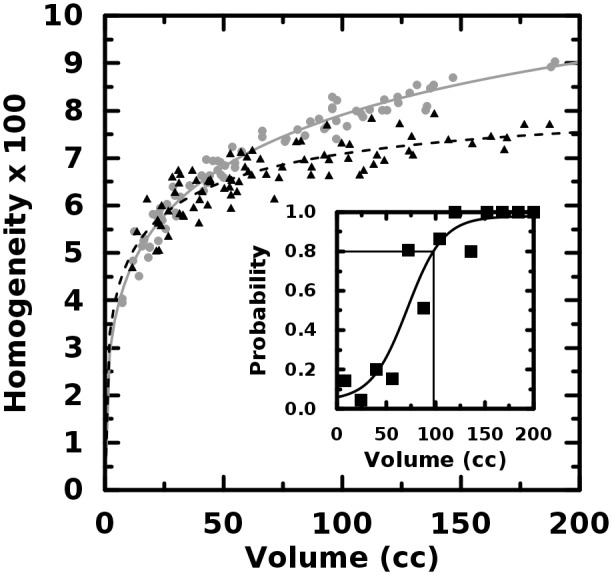
Homogeneity plotted versus object volume. The volume where homogeneity correctly discriminates homogeneous objects (gray, circles) from heterogeneous ones (black, triangles) is slightly less than for dissimilarity.

In the case of the image dissimilarity—or, it’s inverse correlate, the homogeneity—the quantifier value for the heterogeneous object was within 13% of that of the homogeneous object for the first 75% supermajority of object pairings. The conclusion is that small objects differing only in the second order may not exhibit large differences in GLCM-based texture quantifier value. Furthermore, the differences themselves can be seen in Figs. 3 and 4 to scale with volume. This result is illustrated in the figure insets where the probability that the larger quantifier value correctly identifies object type is plotted versus volume. Because of the different volume scaling exhibited by the distinct object types, it is *possible*, for example, for a homogeneous object to exhibit more dissimilarity than its less smooth, more heterogeneous conjugate. It is crucial to see that the trend curves cross and thus the very meaning of “greater value” changes with object volume. Furthermore, despite the strong inverse correlation, the sensitivity of the dissimilarity is less than that of homogeneity. This may be seen from the figure insets where the solid lines indicate the point at which the quantifier value correctly discriminates greater second-order heterogeneity with the 80% probability often employed by clinicians. Because this volume is less for the homogeneity, we expect it will outperform its inverse correlate in distinguishing object pairs, however, it is still larger than many typical clinical volumes. We discuss in a later section why these potentially counter-intuitive results are reasonable and how they likely, profoundly, impact clinical heterogeneity studies.

The generally poor power of the texture quantifiers to discern the isolated second-order effects coupled with the observation of volume scaling distinct to object type, motivated us to implement a more well-established quantifier of local variation. We first define the mean-field gray level <*G*(*x*, *y*, *z*)> as the weighted average of all nonzero gray levels in the 26-connected nearest neighborhood centered at object coordinate (*x*, *y*, *z*). Each gray level in the average is weighted by the reciprocal of the distance between (*x*, *y*, *z*) and the neighbor. The absolute difference in gray level from the mean field is summed over every voxel in the three-dimensional object. That is, we define the mean-field energy for the object to be

$$E\equiv \sum_{x,y,z}|g(x,y,z)-\langle G(x,y,z)\rangle |$$ (1)

**Fig 5:**
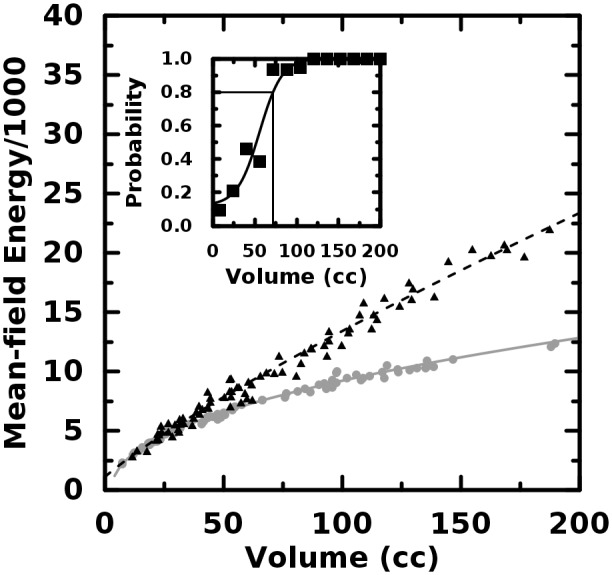
Mean field energy plotted versus object volume. The volume where the mean-field energy correctly discriminates homogeneous objects (gray, circles) from heterogeneous ones (black, triangles) is less than for homogeneity.

Although we are unaware of any published use of *E* as a measure of uptake heterogeneity in FDG-PET images, it is hardly a new quantifier as it is ubiquitous to the field of statistical physics and similar, at least in spirit, to Laws’ measures [34] and to quantifiers based upon neighborhood gray tone difference matrices [20]. It should be clear from Equation 1 that *E* increases with increasing tumor volume. We discuss the potential uses and caveats of *E* as an image heterogeneity quantifier in later section but here note that the difference in volume scaling between the object types is stark (Fig. 5) and the interquartile range of absolute percent differences in *E* is 34%—far greater than that for the other quantifiers. In the figure inset, it is seen that at very large volumes, *E* is always greater for the object with higher second-order heterogeneity. At a volume of 72 cc, *E* is greater with 0.80 probability. This volume is less than for the dissimilarity or homogeneity. Additionally, some computational concerns discussed in a later section *might* make *E* the better quantifier for clinical heterogeneity studies.

### Clinical Image Data

Trends similar to those for the simulated data are summarized in Table 2 for the clinical data that have undergone equal probability quantization. There, it is again seen that the texture quantifiers correlate strongly with volume. It is also of note that none of these quantifiers proved predictive of treatment response via traditional ROC analysis. Additionally, although each of the quantifiers is, in essence, a surrogate for tumor volume, it may not be assumed that the quantifiers are interchangeable [15]. For example, the results of Kendall’s test of rank correlation with the dissimilarity implies that quantifier ranks patients in a very different heterogeneity order than does, the entropy (*τ* = -0.59), the homogeneity (*τ* = -0.69), or the mean-field energy (*τ* = -0.40). We repeated the above analysis for for image data that had *first* been transformed to 8-bits (i.e., 256 gray levels) before quantization or further analysis. From the comparable rank correlations with volume seen in Table 2, it is clear that, as expected, the impact of zero-order (size) effects persist through even drastic reductions bit-depth. Additionally, the predictive capacity does not change appreciably for any quantifier computed upon the quantized image data.

**Table 2:** Texture Quantifiers Applied to Clinical Data.

		Energy	Entropy	Dissimilarity	Homogeneity	Mean-field Energy
15-bit image data	τ	-0.86	0.86	-0.54	0.69	0.78
	AUC	0.50	0.51	0.56	0.54	0.48
8-bit image data	τ	-0.85	0.85	-0.54	0.54	0.79
	AUC	0.50	0.50	0.56	0.55	0.47

Here, τ is Kendall’s measure of rank correlation with tumor volume and AUC is the area under the receiver-operating curve for binary treatment response.

In a second effort to isolate second-order spatial effects from low-order sampling and distribution effects, we sought to compare tumors exhibiting similar low-order properties. First, neither tumor should be so small as to be ambiguously defined. For our cervical cancer data, we set the minimum meaningful volume to 5 cc. Next, because of the observed strong dependence texture quantifiers have upon volume, we demand that comparable tumors be of similar volume. Last, we demand that the gray-level distributions be similar for both tumors. We assessed this via the two-sample Kolomogorov-Smirov test which yields a probability *p* that two samples are drawn from distributions having identical cumulative distribution functions. We thus defined a “low-order similarity” index

$$\psi \equiv \sqrt[{3}]{p(1-\frac{5\text{cc}}{min(V_{1},V_{2})})(1-\frac{\left|V_{1}-V_{2}\right|}{max(V_{1},V_{2})})}$$ (2)

where *V*_*1*_ and *V*_*2*_ are the volumes (in cc) of the tumors to be compared. We computed this index for every possible pairing of our 85 clinical patients before equal-probability quantization was applied and selected only those pairs where ψ > 0.8 for further analysis. This left 20 pairs for which it is plausible that second-order effects might be discernible. Of those 20 pairs, 4 comprised patients with differing treatment response. It is remarkable that, for our data, the supermajority of similar pairs were not particularly large; the third quartile volume is only 32 cc.

For each similarity pair, we computed all quantifiers on quantized image data as before. One key observation is that the energy—for which the IQR of absolute percent difference was only 2% for the simulated images—was 25% for the paired clinical data. This implies that controls of low-order effects imposed via the similarity index were insufficient to counteract the extreme sensitivity of the energy to whatever low-order differences persist. We contrast this to the dissimilarity and homogeneity where the IQR of the absolute percent differences (11%, 16%, respectively) for our clinical data paired via Equation 2 is comparable to those of the simulated images. While this implies that it is plausible that second-order differences within clinical pairs might be measurable, the fact that each difference is greater also implies that the low-order similarity (*ψ*) may need to be set even higher than 0.8 in order to truly isolate second-order effects in clinical studies.

We assume that if a second-order heterogeneity quantifier is of prognostic value, then the quantifier value should be (approximately) equal for pairs with identical treatment response and decidedly unequal for pairs with differing response. We investigated this in two ways. First, we defined equivalence of quantifier value as any pairwise absolute difference less than that of the Freedman-Diaconis bin size for all unique pairwise absolute differences. We formed the 2x2 contingency table with outcome difference and quantifier difference. These results are summarized in Table 3 where it is seen from Cramer’s *ϕ* that no quantifier correlates with treatment response. Second, because each quantifier depends upon volume—even within our relatively narrow bands of tumor similarity—we also analyzed the detrended pairwise absolute differences. For each quantifier, we computed *f(V)*, the least-squares non-linear fit of all values in our clinical data set. These *f(V)* (not shown) are similar in functional form and fit to the trend curves shown in Figs. 3–5 for the simulated data. We subtracted *f(V)* from each quantifier value at each *V*. The detrended absolute differences between paired patients were then tested as a predictor of outcome via ROC analysis. Again, we found that no quantifier predicted treatment response (Table 3). We repeated the above analysis for a new set of clinical similarity pairs determined from the original image data that had *first* been transformed to 8-bits before quantization or tests via Equation 2. This gave us 31 similarity pairs with 9 comprising patients of different treatment response. The increase in similarities is expected given that reduction of bit depth smooths the input gray-level histograms. As seen in Table 3, the ROC analysis again indicates no reliable predictive capacity for any of the quantifiers tested, however, the AUCs are different from those of the previous case for the entropy and homogeneity. The implication is that the statistic often used to determine the predictive capacity of clinical heterogeneity statistics depends upon the pre-quantization bit-depth of the input images.

**Table 3:** Texture Quantifiers Applied to Similar Clinical Pairs.

		Energy	Entropy	Dissimilarity	Homogeneity	Mean-field Energy
15-bit image data	ϕ	0.063	-0.055	0.085	0.085	-0.064
	AUC	0.53	0.39	0.66	0.55	0.64
8-bit image data	ϕ	0.087	-0.18	0.12	0.32	-0.39
	AUC	0.50	0.46	0.66	0.70	0.62

Note that here Cramer’s *ϕ* is derived from raw quantifier values whereas the AUC was derived from volume-detrended quantifier values.

## Discussion

Analysis of image texture as a technique for quantifying and discerning pre-defined regions-of-interest (ROIs) has been proven effective in diverse applications such as remote sensing [17], histopathology [23], facial recognition [24], autonomous vehicle guidance [25], handwriting analysis [26] and tree species identification [27]. Common to all of these examples, however, is that the ROIs compared tend to be larger and/or of higher resolution than those found in FDG-PET images of tumors. As we have illustrated for both theoretical and clinical cases, texture quantifiers are dominated first by ROI size. This is a kind of “zero-order” effect: before any higher-order differences *can* measured, the ROI must be large enough such that the features to be measured have space enough to manifest. In other words, one cannot expect to find a size *L* feature in a region of size less than *L*. Thus, all quantifiers of spatial arrangement must depend somewhat on region size [16, 17, 20]. We therefore predict that the dependence upon tumor volume is even greater for quantifiers of increasing sophistication because the number of occurrences of larger features (e.g., distant pairs, runs or zones) must be fewer at smaller volumes. It is therefore prudent to presume that sophisticated texture quantifiers may not have accrued enough feature samples at small volumes as to make any meaningful comparison between disparate tumor sizes. An apt analogy would be attempting to prove a suspect coin to be unfair by flipping it only twice. In both scenarios, the behavior sought simply cannot be measured until many samples are observed. The presence of zero-order (size) effects implies that small tumors could be of the exact same biology as large ones yet this biology would be untestable via FDG-PET texture analysis because it always yields different quantifier values for disparate volumes.

We made some attempt to compute the volume at which comparisons may be meaningful. However, we must stress two critical issues with this. First, every such assessment must address a specific task. That is, each quantifier in question must be tested for each type of image data. Even then, the minimum volume estimate may not be appropriate. We constructed image data with object size, shape and gray level distribution which are consistent with our clinical data. However, that consistency alone implies neither that we’ve discovered some widely applicable rule nor that we’ve computed the precise minimum volume for cervical cancer data. At best, we can only say that it seems likely that the minimum volume is large, maybe so large as to exclude the majority of our clinical data. Absent of some rigorous analytic proof otherwise, it should never be presumed that any minimum volume is applicable for all quantifiers or all data types. Secondly, the numeric value of volume is not important but instead the number of voxels is. This is because PET scanner settings differ from study to study. For our simulated images, we observed that 72 cc yielded 80% probability that the mean-field energy could correctly distinguish second-order heterogeneity. This corresponded to a minimum sampling of 1125 voxels; a substantially larger estimate than one published previously [22]. It is also important to note that these concerns about post-hoc *image* sampling are distinct from those regarding pre-reconstruction positron counting or from the theoretical computation of PET spatial resolution in general. Our concerns derive from the analysis of the image data clinicians typically have in-hand and thus remain germane even after the effects of specific scanner settings or image reconstruction techniques are considered.

The size and shape of the voxels themselves likely impacts clinical heterogeneity studies. Consider, for example, two tumors of equal volume *V*. If the first is assayed via a scanner set to voxel size *v* while the second is assayed via voxel size *γv* where γ > 1, then the second must comprise fewer total voxels (i.e., samples). Thus, the rate that the second scanner approaches adequate sampling—whatever that adequacy may be—is necessarily different from that of the first scanner. Thus, the *en masse* analysis of image data from scanners with vastly different voxel size likely includes this underlying variance. Additionally, it is also reasonable to presume that differences in voxel shape could introduce still more variance. Consider that some institutions set their PET scanners such that the trans-axial resolution differs from the planar resolution. That is, the voxels might be longer along the *z*-axis than in either the *x*- or *y*-axes. In such a case, the relative distance between voxels is not equivalent in all directions. However, this is precisely what is tacitly assumed in every clinical image texture analysis we have thus far encountered. Recall that due to PET physics, intensities within neighboring voxels necessarily are correlated and the strength of that correlation decreases non-linearly with distance. If texture quantifiers which, by definition, measure only *relative* voxel juxtaposition are extended into three dimensions, but the new dimension is not equivalent to the others, that extension must introduce additional variance. In other words, if the voxels aren’t cubical, the biological meaning of heterogeneity in one axial plane is not the same as in another. While here we make no attempt whatsoever to compute the ultimate impact of voxel size and shape on clinical heterogeneity studies, it seems prudent to consider that these effects could be an additional source of discrepancy between previously published studies.

A more certain aspect about quantifier dependence upon volume is the observation that the trend curves for the differing object types cross. While we cannot claim from the simulated images that this phenomenon must happen in all clinical data, the very possibility raises serious concern. If, for example, one seeks to declare tumor *A* more spatially heterogeneous than tumor *B*, one must first declare the direction of the effect. In other words, the implication of measuring a larger quantifier value must be established first. In the arbitrarily chosen case of the homogeneity, a larger value implies more homogeneity if the volume is greater than 27 cc (for our simulated data). For smaller volumes, on average, a larger value implies *less* homogeneity. Again, we emphasize that we do not claim to have computed this crossover for any other data set clinical or otherwise. However, we do now know it *possible* for the texture quantifiers employed to exhibit such trend-crossing behavior. Coupled with the fact that this crossover point cannot be known for clinical data *a priori*, it is difficult to see how analysis of tumor volumes anywhere near a potential crossover point could be done reliably. The likely source of the distinct scaling with volume are the gray-level distributions themselves. As seen in Table 1, the dissimilarity and homogeneity each depend upon the absolute gray-level difference, |*m*−*n*|. This means that co-occurrences between disparate gray levels are weighted more heavily than co-occurrences between similar levels. This alone could explain why the dissimilarity and homogeneity exhibit such different scaling for the different object types while entropy or energy do not. It also explains why the homogeneity, which scales as 1/ |*m*−*n*|, appears more sensitive than the dissimilarity. If, as is reasonable to assume from above and from Ref. [22], gray level frequency decreases with level brightness (on average), then extremely different co-occurrences are relatively rare. Furthermore, because the differently arranged object types necessarily differ in rate of *any* co-occurrence, it seems reasonable that a quantifier dependent on |;*m*−*n*|; could regress at different rates for distinct objects sharing the same gray level distribution. This is why we analyzed our clinical data via absolute differences and made no attempt to attach verbal meaning (e.g., “more heterogeneous”) to the binary response variable.

To better appreciate the implications of first-order effects, consider that because gray levels *m*, *n* correspond to measurable values such as the SUV or the radioactivity, it is the |*m*−*n*| term which gives a texture quantifier physical meaning. Thus, |*m*−*n*| -dependent quantifiers computed from differently shaped histograms or histograms on different scales can have different physical meaning. This permits the possibility that a particular clinically interesting juxtaposition differing by |*m*−*n*| in one patient may be considerably less probable in another patient. This bias is not necessarily removed by simply re-normalizing the measured SUV range to an arbitrary gray-level bit depth (e.g., 2^8^ levels). Depending on the ranges observed, typical SUV distribution peaks and tails can persist even into severe bit depth reductions. For this reason, using equal-probability quantizing might reduce the ambiguity and effects of comparing drastically different gray-level distributions. This is done via a mapping of the gray levels according to count and rank, not magnitude. Thus, metrics computed upon the quantized levels are less sensitive to gray level outliers. It should be noted that such histogram equalization indeed was prescribed by Haralick et al. in their seminal work describing texture quantifiers [17].

One concern relevant to FDG-PET data is the sparsity of equalized gray-level histograms. A typical PET scanner is capable of outputting 15-bit images comprising 2^15^ gray levels but the number of differing SUV levels observed for any one patient is typically many, many fewer than 2^15^. Therefore, when the typical (for our data) *N*~10^2^ to 10^3^ unique gray levels are quantized to the entire available bit depth, the resulting histogram is sparse; typically, the levels are distant and each of frequency equal to only one or two. Although this sparseness alone does not preclude use of texture quantifiers, it might be desirable for clinical applications to employ a more intuitive gray-level scale. One might then apply a wider, independently determined histogram bin size—such as the Freedman-Diaconis bin size, for example—to the quantized levels. The result (for our data) is an approximately flat histogram over the entire 15-bit depth but with relatively few unique levels (bins). This might be desirable in cases where clinically relevant changes in SUV are large and thus do not require many distinct gray-levels. For example, if one finds that a spectrum of about 64 distinct SUV levels are relevant to a particular clinical task or study [5], then perhaps quantization to 6-bits might be sufficient. It should be clear, however, from Table 3 that the choice of bit depth strongly affects the final output of clinical heterogeneity studies. This may be seen via the increase in AUC for the homogeneity from the 15-bit to the 8-bit image data input. We note, however, that this AUC = 0.70 is not impressively great given that, for our patient population, one has a 65% chance of correctly guessing treatment response by chance alone. Here, a higher area under the ROC curves (AUC) does not necessarily indicate that one bit depth should be preferred over another. The simpler explanation of varying AUC values is that low-order effects persist through bit depth reduction and thus any discerning capability of the texture quantifiers is actually that of the low-order distribution properties being unequally affected by that reduction. While low-order properties may indeed be clinically useful, they absolutely do not represent the chiefly spatial qualities ostensibly sought when employing a second-order texture quantifier. This implies that the biological meaning assigned to intra-tumor heterogeneity can itself depend upon the *choice* of bit depth. We therefore suggest that equal-probability quantization be applied and that the appropriate bit depth always be determined independently via sound physical reasoning and never chosen arbitrarily.

A bonus of applying a wider bin width to the quantized gray levels is that computation time is reduced via fewer elements appearing in the GLCM. We note that severe reduction of bit depth necessarily reduces the number of significant digits for texture quantifiers we mention. For our work, we used the sparse histograms comprising on the order of 10^2^ to 10^3^ levels as originally output by the quantizing algorithm. We feel that the main point of equal-probability quantization in the present context is the elimination of the arbitrariness of gray-level scale. We note, however, that even though histogram equalization described above diminishes first-order effects, the zero-order effects described above persist.

Histogram equalization augments visual contrast near histogram minima and diminishes it near histogram maxima [16]. Thus, equalization necessarily alters the heterogeneity of the image. However, because our task is to quantify the heterogeneity visually observed by clinicians, we take this as further evidence that *a priori* contrast enhancement is appropriate. In order to better assess the effect contrast enhancement has on clinical studies, some estimation of the “size” of the heterogeneity sought must be made. That is, one must define what constitutes a small image feature versus a large one. However, that is precisely what we seek to learn by applying spatial quantifiers to FDG-PET images. Pragmatically, the appropriate spatial scale may be moot. With current PET technology, we may only be able to assess nearest-neighbor co-occurrences because statistically rigorous analysis of farther ones (i.e., larger features) generally is not supported by the small sample sizes (tumor volumes).

The strong dependence of second-order quantifiers upon low-order effects yields two clinically important corollaries. First, given that quantifier magnitudes for purely second-order (arrangement) differences are so small in comparison to those resulting from low-order (size, distribution) differences, it is logically possible that two tumors could be of distinctly different spatial biology yet this biology would be untestable via FDG-PET texture analysis. The second corollary is a general hypothesis as to why clinical studies of prognostic FDG uptake heterogeneity have yielded such inconsistent results: the quantifiers likely are measuring heterogeneity of the patient population. In the present context, population heterogeneity might occur via the pooled analysis of patients with largely different tumor volumes, stages, histologies or treatments. We know that many texture quantifiers are monotonically dependent on volume. For many types of cancer studies, large tumors tend to be much rarer than smaller ones. Taken together, we must then expect the texture quantifier values to be asymmetrically distributed as well for many populations studied. Further evidence of this is the fact that ROC analysis of the pooled clinical data yielded different prognostic results than that of the paired clinical data. The implication is that ROC results can be tuned by changing the proportions of volumes or SUV distributions within the patient population. We therefore suggest that future studies of spatial FDG-uptake heterogeneity be of patient populations which are themselves as homogeneous as possible. When that is not logistically possible, or when low-order effects upon a chosen quantifier are unclear, perhaps patients of similar low-order properties could be identified via Equation 2 then analyzed against each other as we did in the present work.

Image analysis via texture quantifiers can be computationally intensive for two main reasons. If a measured range has *N* gray levels, then the GLCM has *N* ^*2*^ elements. Additionally, the GLCMs are innately directional. Thus, each time a new direction is considered, another *N* ^*2*^ elements must be computed. For FDG-PET data, there is no standard for choosing the directions or for how to combine quantifiers calculated for different directions. Furthermore, any directional system imposed upon the image set is always arbitrarily orientated relative to the tumor within each patient. In other words, there is absolutely no good reason to assume that a quantifier computed along the vector direction <1, 0, 0> in one image set has the same meaning in another image set. Some of this ambiguity and computational burden might be avoided by employing the mean-field energy, *E*, as a texture quantifier (Equation 1). That quantifier assesses spatial variations in the sense that it accrues differences between neighboring voxels but it remains non-directional in that equidistant neighbors are treated simultaneously and identically. Furthermore, the computational order scales linearly with object size, not quadratically with gray level range. Thus, for typical clinical volumes, *E* can be computed very quickly on a common desktop computer. For larger volumes, we found that *E* was able to perfectly discriminate the heterogenous objects from the homogenous ones. For our clinical pairings, however, ROC analysis showed no such impressive capability. While this result could imply that second-order quantification of uptake heterogeneity has nothing to do with treatment response, the simpler explanation is that the volumes analyzed (<≈50 cc) were not large enough for *E* to be effective.

## Conclusion

We have shown that zero-order (size) and first-order (distribution) effects likely dominate second-order (arrangement) measures of FDG-PET uptake heterogeneity in clinical studies. The implication is that low-order effects can create apparent second-order heterogeneity differences where none actually exist and also can mask genuine differences. Furthermore, we’ve identified a plausible mechanism by which it is possible that the very meaning of a second-order difference can change as a function of tumor volume. These facts motivate our argument that image texture analysis of typical FDG-PET images is strongly influenced by heterogeneity of the patient population itself. We therefore suggested a method for identifying patients with similar low-order properties. Our results showed that similar-pair analysis of clinical data yields different—and arguably more believable—results than does the current practice of pooling images from patients with disparate tumor volumes, stages, histologies and SUV ranges. Thus, similar-pair analysis may be a more promising technique for studying the prognostic potential of observed FDG uptake heterogeneity. Whatever the case, it is clear from our results that analysis of typical FDG-PET images via texture quantifiers is a complicated task rife with research opportunities and pitfalls.

## Supporting Information

S1 Object Coordinates Simulated Compressed text file of (*x*, *y*, *z*, *g)* coordinates of the objects created as described in the Methods section. The Cartesian coordinates (*x*, *y*, *z)* are given in pixels where *x* and *y* are the column and row, respectively, relative to the top-right corner of the image which is defined to be (0,0). The *z* value is then the image index within the sequence of images for a single object. It is assumed that each The gray values *g* are given on an 8-bit, 256 shade scale.
(ZIP)

S1 Object Coordinates Clinical Compressed text file of (*x*, *y*, *z*, *g)* coordinates of the clinical data analyzed as described in the Methods section. The Cartesian coordinates (*x*, *y*, *z)* are given in pixels relative to the top-right corner of the image and the gray values *g* are given on a 15-bit, 32768 shade scale.
(ZIP)
